# Evaluation of read count based RNAseq analysis methods

**DOI:** 10.1186/1471-2164-14-S8-S2

**Published:** 2013-12-09

**Authors:** Yan Guo, Chung-I Li, Fei Ye, Yu Shyr

**Affiliations:** 1 Center for Quantitative Sciences, Vanderbilt University, Nashville, TN, USA

**Keywords:** RNAseq, expression, DESeq, DEGseq, edger, NBPSeq, TSPM, baySeq

## Abstract

**Background:**

RNAseq technology is replacing microarray technology as the tool of choice for gene expression profiling. While providing much richer data than microarray, analysis of RNAseq data has been much more challenging. To date, there has not been a consensus on the best approach for conducting robust RNAseq analysis.

**Results:**

In this study, we designed a thorough experiment to evaluate six read count-based RNAseq analysis methods (DESeq, DEGseq, edgeR, NBPSeq, TSPM and baySeq) using both real and simulated data. We found the six methods produce similar fold changes and reasonable overlapping of differentially expressed genes based on p-values. However, all six methods suffer from over-sensitivity.

**Conclusions:**

Based on the evaluation of runtime using real data and area under the receiver operating characteristic curve (AUC-ROC) using simulated data, we found that edgeR achieves a better balance between speed and accuracy than the other methods.

## Background

The process by which information from a gene is used in the synthesis of a functional gene product is called gene expression. The process of gene expression is used by all known life including eukaryotes, prokaryotes, and viruses to generate the macromolecular machinery for life. Gene expression analysis is essential for biomedical research. The introduction of microarray technology has helped biomedical research make significant advances in the last decade by allowing high-throughput gene expression screening on all known genes. Recently, the introduction of RNAseq technology has had a revolutionary impact on the field of expression research. RNAseq refers to the use of next-generation sequencing (NGS) technologies to sequence cDNA in order to get information about a sample's RNA content. Compared to microarray technology, the RNAseq method offers several distinct advantages. First, the detection range of RNAseq is not limited to a set of predetermined probes as with microarray technology, so RNAseq is capable of identifying new genes. Second, the resolution of a microarray is limited to the gene level for most arrays and the exon level for specially designed exon arrays. On the other hand, RNAseq can detect expression at the gene, exon, transcript, and coding DNA sequence (CDS) levels. Finally and most importantly, RNAseq can detect structural variants such as alternative splicing and gene fusion. With the maturity of NGS technologies, the price of RNAseq has become comparable to microarrays. Many researchers have predicted the inevitable replacement of microarray by RNAseq [1-3] based on the competitive price and additional genomic information contained in RNAseq data.

With the rich genomic information RNAseq technology brings, it also comes with complication in the analysis phase. To date, the research community has not yet come to a consensus on the best approach for analyzing RNAseq data. The most popular normalization method for microarray data is Robust Multi-array Average (RMA) [4], a form of quantile normalization. For RNAseq, one of the popular normalization methods is Reads per Kilobase per Million mapped reads (RPKM) [5] or Fragments Per Kilobase of transcript per Million mapped reads (FPKM) [6]. The RPKM of a gene is computed as follows: $RPKM=10^{9}\times \frac{C}{N*L}$, where  C is the number of reads mapped to the gene,  N is the total number of reads mapped to all genes, and  L is the length of the gene. FPKM is computed similarly to RPKM, except it accounts for the scenario in which only 1 end of a paired-end read is mapped. In addition to RPKM and FPKM, other read count methods based on Possion, negative binomial, and Bayesian methods also exist. Each method has unique strengths and weaknesses. In this study, we focus on read count-based methods and systematically evaluate 6 RNAseq R packages including DESeq [7], DEGseq [8], edgeR [9], baySeq [10], TSPM [11] and NBPSeq [12] using both real and simulated data. BaySeq is considered an empirical Bayes approach to detect patterns of differential expression, DESeq and NBPSeq are based on a negative binomial model, DEGseq and TSPM are based on a Poisson model, and edgeR uses empirical Bayes estimation and exact tests based on the negative binomial.

## Method

The real RNAseq datasets were selected from The Cancer Genome Atlas (TCGA) [13,14]. TCGA is a massive, comprehensive, and collaborative project to catalogue genomic data for over 20 types of cancers by the National Cancer Institute (NCI), the National Human Genome Research Institute (NHGRI), and 27 institutes and centers of the National Institute of Health (NIH). Gene expression profiling by RNAseq is one of the major components of genomic data collected by TCGA. Breast cancer is the only cancer type in TCGA that collected expression data on a large quantity of tumor-normal paired samples. Thus we selected breast cancer tumor-normal paired data (53 pairs) as our primary source of real RNAseq data. Differentially expressed genes between tumor and normal were identified using all six methods at the significance level of 0.05 with Benjamin-Hochberg False discovery rate (BH FDR) adjustment. To evaluate the consistency between the six methods, we computed pairwise Spearman's correlations as well as intraclass correlation (ICC) for fold change values of all genes, along with the corresponding p-values. In addition, we evaluated each method's running time.

For each gene, the count is drawn from the negative binomial distribution with the mean and dispersion parameters estimated from the TCGA breast cancer dataset. For a given gene *m*, the fold change is calculated as ${\left(\rho_{m}+\rho_{*}\right)}^{d_{m}}$ where $\rho_{m}$ is drawn from the gamma distribution with shape parameter 0.87 and rate parameter 1.36 (parameters are estimated from the TCGA breast cancer dataset), $\rho_{*}$ is the lower bound of fold change, and

$$d_{m}=\left\{\begin{array}{c} 1\;\;\;\;\;\;\;\;\;,\text{if}\;\text{gene}\;\text{m}\;\text{is}\;\text{upregulated}\\ -1\;\;\;,\text{if}\;\text{gene}\;\text{m}\;\text{is}\;\text{downregulated}\\ 0\;\;\;\;\;\;\;\;\;\;\;\;\;\;\;\;\;\;\;\;,\text{otherwise} \end{array}\right).$$

We evaluated the methods using datasets simulated to present different scenarios corresponding to a given combination of the following parameters: sample size (5 or 10), proportion of differentially expressed genes (5% or 10%), ratio of up-regulated vs. down-regulated (1:1 or 3:1), lower bound of fold change (1.5 or 1.1), and lower bound of depth (5 or 1). A total of the seven most representative scenarios are shown in Table 1. For each scenario, 30 datasets were simulated from a negative binomial distribution. To evaluate the performance of the six methods, we calculated the number of genes that are significantly differentially expressed (*N_S_*), the rate of false positives (*FPR*), and the rate of true positives (*TPR*) across 30 simulation datasets for each scenario. False discovery rate (*FDR*) at levels of 0.1, 0.05 and 0.01 were used as the cutoffs. In order to measure the overall performance, we also computed the area under the curve (AUC) across 30 simulation datasets under each scenario.

**Table 1:** Scenarios in the simulation comparison study

Scenario	Sample size	DE (%)	Up/down	Lower bound of fold change	Lower bound of depth
I	10	10	1:1	1.5	5
II	2	10	1:1	1.5	5
III	10	5	1:1	1.5	5
IV	10	10	3:1	1.5	5
V	10	10	1:1	1.1	5
VI	10	10	1:1	1.5	1
VII	2	10	1:1	1.1	1

## Results and discussion

**Figure 1:**
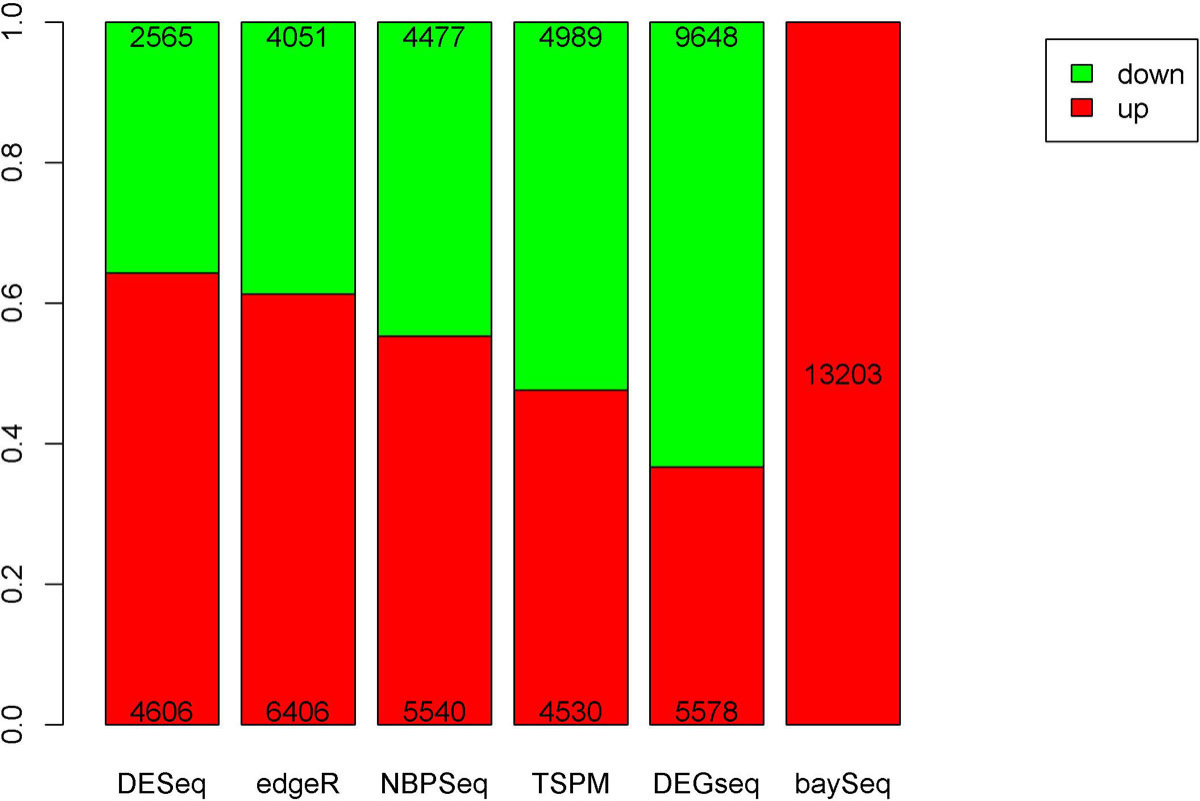
**Number of significantly differentially expressed up-regulated and down-regulated genes for each method**.

We performed differential expression analysis on the 53 tumor-normal paired samples from TCGA using all six methods. Out of 16,146 genes, DEGseq identified the most number of significant genes at 15,226. That is, 94.3% of all genes were identified by DEGseq as statistically significantly differentially expressed between tumor and normal, which implies that DEGseq is over-sensitive. DESeq on the other hand identified the least number of differentially expressed genes with 7,171, which is still too many to be biologically plausible. NBPSeq, edgeR, TSPM, and baySeq identified 10,017, 10,457, 9,519, and 13,203 differentially expressed genes respectively (Figure 1). In terms of up-regulated genes vs. down-regulated genes, DESeq, edgeR, and NBPSeq identified more up-regulated genes, while TSPM and DEGseq identified more down-regulated genes. BaySeq does not provide fold change or test statistics, thus no direction of gene expression change can be determined through p-value alone. A unique characteristic associated with baySeq is that there is a randomization factor built into its computation model. Using baySeq to analyze the same dataset on same parameter settings twice will generate slightly different results. In our scenario, the difference can be as many as 100 genes.

**Figure 2:**
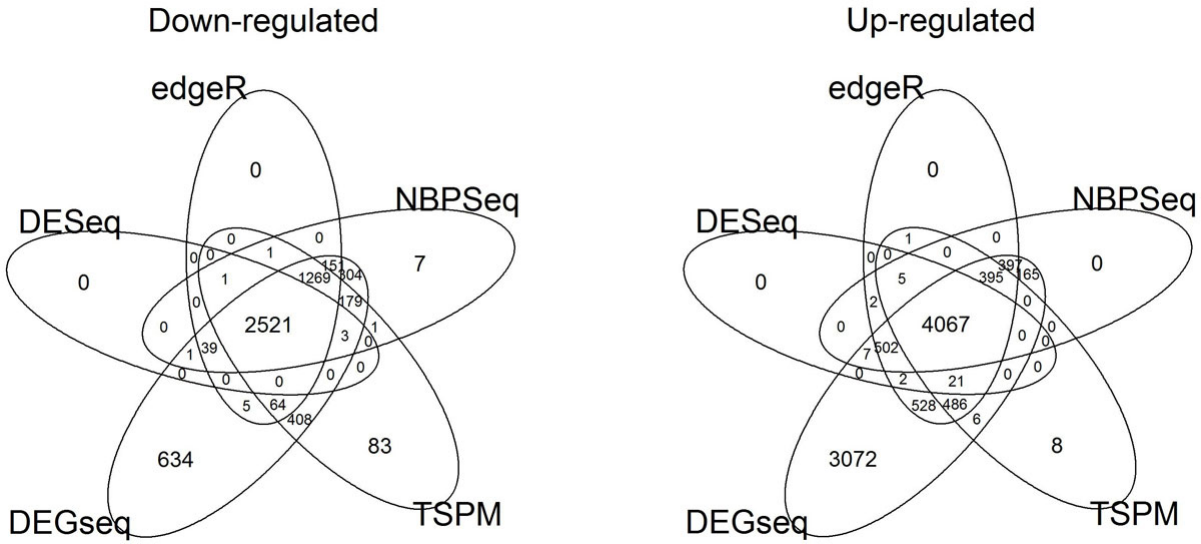
**Venn diagram of the overlap in differentially up-regulated and down-regulated genes among five methods (excluding baySeq)**.

Next we computed the overlap of differentially expressed genes between methods for both up-regulated and down-regulated genes. BaySeq was excluded from this analysis due to its lack of directionality (Figure 2). For down-regulated genes, 2,521 were identified by the rest of five methods, and for up-regulated genes, 4,067 were identified by all five methods. DESeq, edgeR, and NBPSeq observed the fewest singleton genes (defined as genes identified by only 1 method) in terms of both up-regulated and down-regulated genes. TSPM observed a moderate number of singleton genes, and DEGseq observed the most, which is also a reflection of its over-sensitivity.

**Figure 3:**
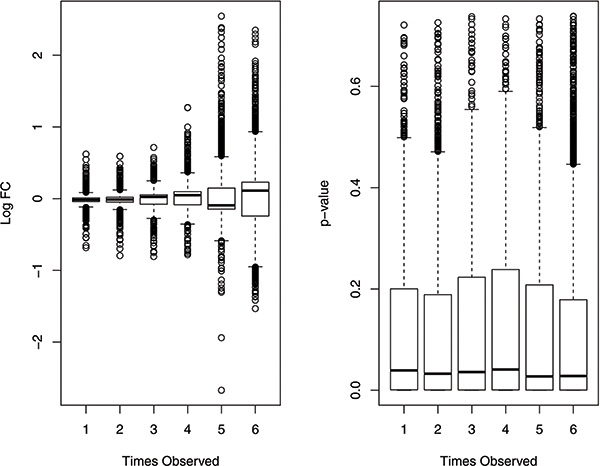
**The relationship of fold change and p-value vs. identification frequency**.

We studied the relationship of fold change and p-value vs. identification frequency of significantly expressed genes detected by the six methods (Figure 3). As expected, genes identified by all six methods had a stronger signal (e.g., a higher fold change value) than genes identified by fewer methods. The singleton genes had the lowest fold change values (Figure 3 left). However, for p-value the pattern was not clear. No significant association between p-value and identification frequency was observed.

**Figure 4:**
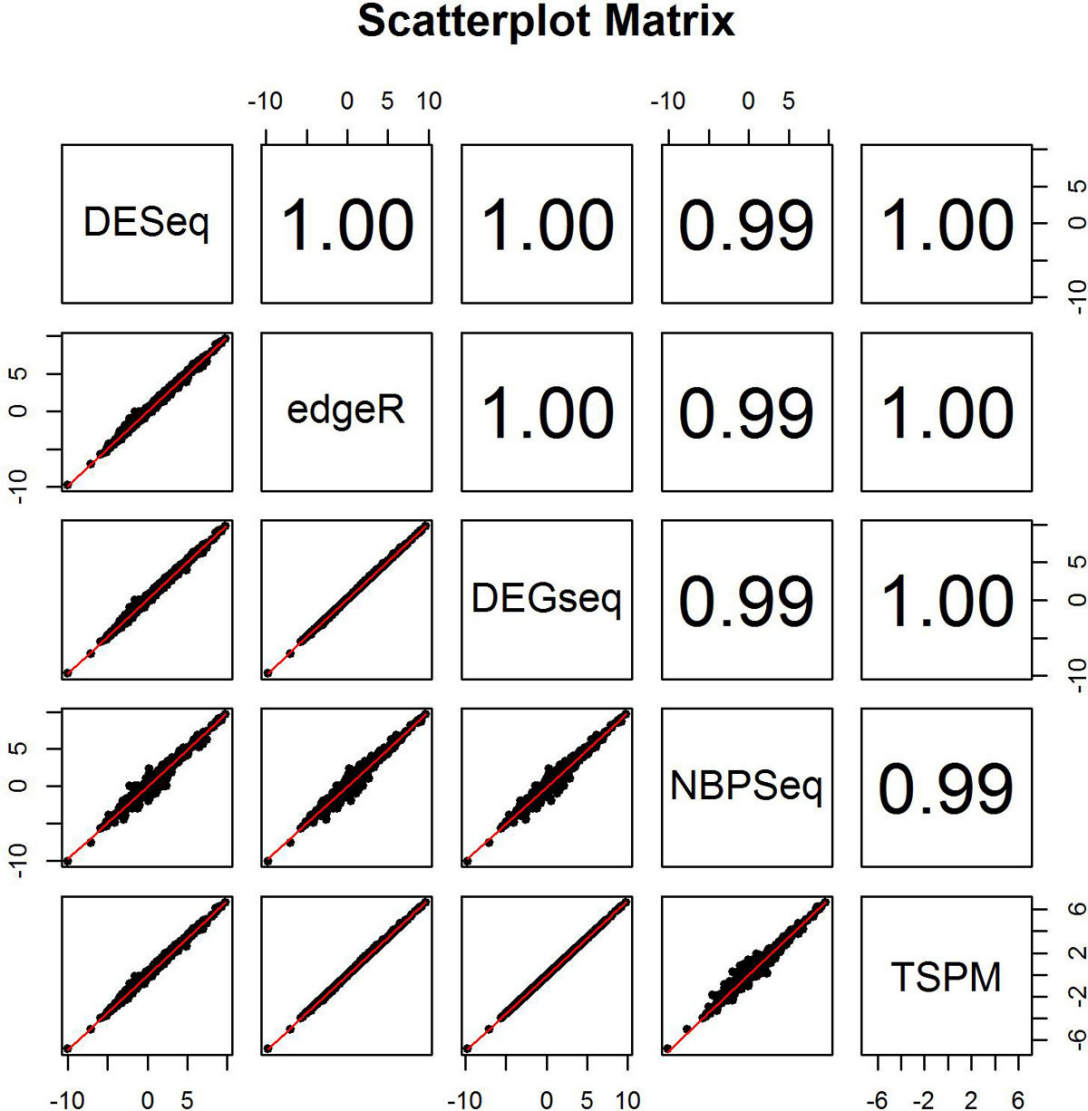
**Pair-wise Spearman's correlation coefficients of fold change computed among six methods**.

**Figure 5:**
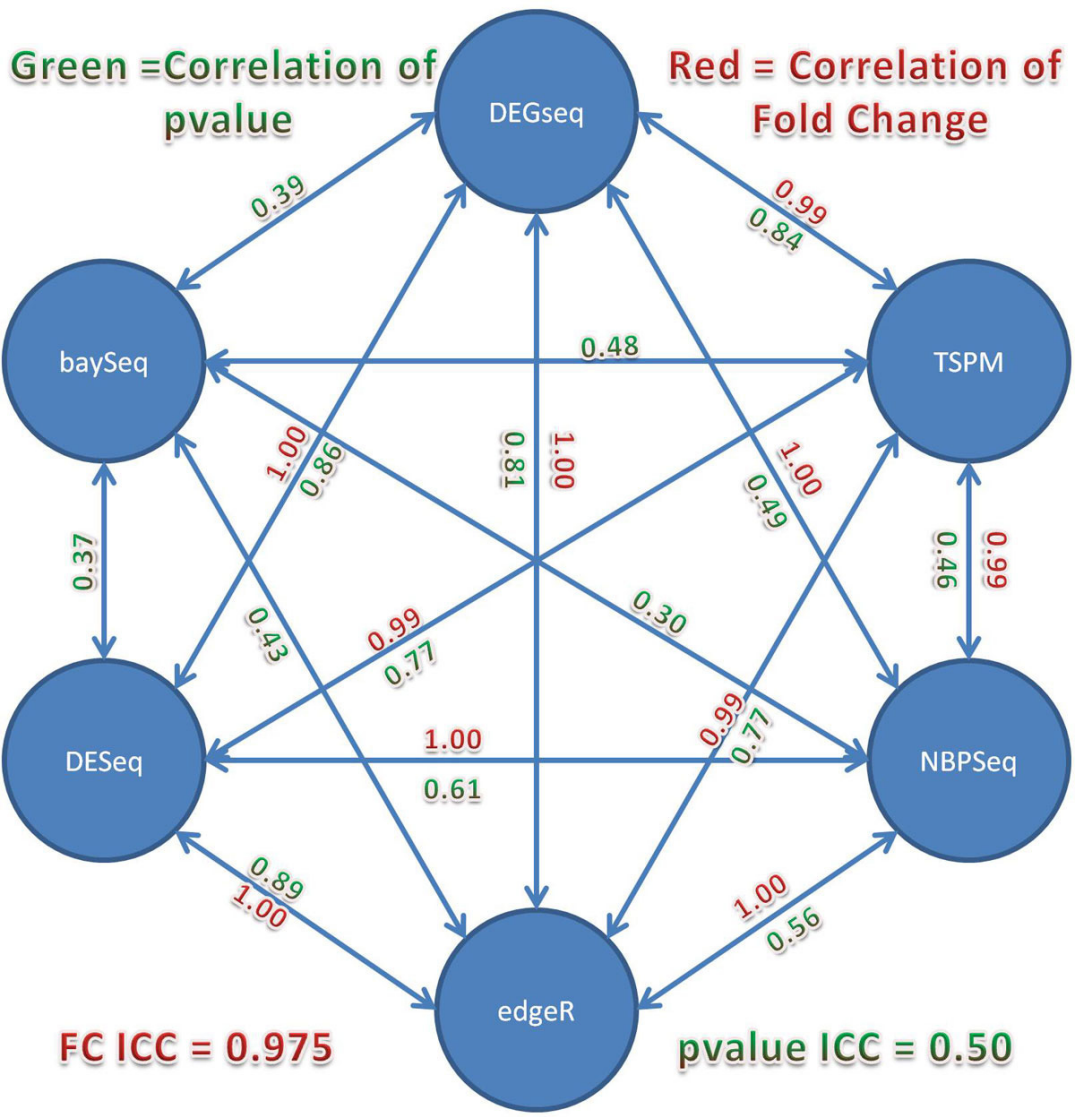
**Intraclass correlation coefficients among six methods using fold change and p-value**.

We also tested the consistency among the six methods at overall significance levels. Pair-wise Spearman's correlation coefficient of fold change values was computed (Figure 4). Most correlation coefficients are very close to 1, which indicates that the six methods are highly consistent in terms of ranking genes according to fold change despite difference in normalization method. We also computed intraclass correlation coefficients (ICC) using both fold change and p-value as well. The ICC based on fold change was 0.975 which strongly suggests that a high degree of agreement among the five methods (excluding baySeq because no fold change can be calculated) as the fold change value is only affected by different normalization techniques used by different methods (Figure 5) However, the ICC for p-value was only 0.50, indicating that these methods have a low agreement in terms of ranking the differentially expressed genes. In addition, baySeq produced the worst p-value correlation with regard to the other methods. To assess accuracy, one has to know a priori which genes are differentially expressed between normal and tumor. Hence, we can only calculate it in a simulation.

For simulated data, we compared the number of genes that are significantly differentially expressed. In contrast to the results from real data, baySeq identified the smallest number of differentially expressed genes, while DEGseq identified largest number of differentially expressed genes, significantly more than other methods (Table 2). DESeq, edgeR and NBPSeq identified similar numbers of differentially expressed genes. The similar performance of DESeq, edgeR, and NBPseq is probably due to the fact that these methods are based on similar principles except the estimation of the dispersion parameter. Among all six methods, baySeq has the smallest FPR, and DEGseq has the largest FPR. This indicates that baySeq was more conservative compared to other methods. Among DESeq, edgeR and NBPSeq, the largest FPR was found by NBPSeq, followed by DESeq or edgeR. In general, the FPR of DESeq was smaller than the FPR of edgeR. For TPR, the largest TPR was found by DEGseq (i.e. it also found too many false positives), followed by TSPM. As expected, DESeq, edgeR and NBPSeq obtained similar results in TPR. After comparing the results under scenarios I and II, we found that sample size has a large effect, especially for TPR. As sample size decreases, *N_S _*decreases, FPR increases, and TPR decreases, respectively. TSPM shows the strongest effects on sample size among the methods. After comparing the results under scenarios I and III, we found that the proportion of differentially expressed genes has a relatively small effect on FPR and TPR for all methods. As the proportion decreases, all of *N_S_*, FPR, and TPR decrease. After comparing the results under scenarios I and IV, we found that the proportions of up-regulated and down-regulated genes have a large effect on *N_S _*and FPR for TSPM and NBPSeq. After comparing the results under scenarios I, V, and VI, we found that the level of treatment effects and depth of reads have small effects for all of the methods under reasonable sample size. Comparing the results under scenarios I and VII, all methods are sensitive to the sample size, level of treatment effects, and depth.

**Table 2:** Performance comparison under different scenarios

		FDR = 0.1			FDR = 0.05			FDR = 0.01		
		
		N_s_	FPR	TPR	N_s_	FPR	TPR	N_s_	FPR	TPR
I	DESeq	759	0.0090	0.6779	679	0.0054	0.6304	550	0.0020	0.5316
	Edger	825	0.0115	0.7216	740	0.0068	0.6799	614	0.0023	0.5934
	DEGseq	8814	0.8735	0.9532	8864	0.8573	0.8573	8415	0.8306	0.9402
	NBPSeq	1167	0.0480	0.7346	972	0.0322	0.6828	723	0.0153	0.5850
	Bayseq	729	0.0060	0.6750	654	0.0027	0.6296	543	0.0006	0.5384
	TSPM	2307	0.1687	0.7893	1815	0.0120	0.7306	1202	0.0663	0.6050
										
II	DESeq	289	0.0153	0.1515	175	0.0096	0.0890	59	0.0037	0.0255
	Edger	239	0.0138	0.1139	130	0.0080	0.0580	38	0.0028	0.0127
	DEGseq	8379	0.8292	0.9162	8215	0.8119	0.9081	7921	0.7801	0.8923
	NBPSeq	308	0.0207	0.1219	194	0.0134	0.0729	70	0.0054	0.0213
	Bayseq	189	0.0070	0.1260	88	0.0029	0.0617	10	0.0003	0.0067
	TSPM	291	0.0262	0.0557	262	0.0234	0.0513	217	0.0191	0.0447
										
III	DESeq	363	0.0056	0.6200	319	0.0034	0.5739	255	0.0013	0.4860
	Edger	401	0.0068	0.6729	354	0.0041	0.6301	284	0.0014	0.5414
	DEGseq	8451	0.8391	0.9598	8288	0.8222	0.9555	8002	0.7925	0.9462
	NBPSeq	567	0.0241	0.6767	474	0.0169	0.6283	346	0.0084	0.5335
	Bayseq	332	0.0031	0.6052	295	0.0015	0.5615	242	0.0004	0.4763
	TSPM	851	0.0529	0.6892	651	0.0354	0.6311	426	0.018	0.5035
										
IV	DESeq	758	0.0090	0.6770	678	0.0055	0.6290	544	0.0019	0.5269
	Edger	828	0.0117	0.7225	739	0.0068	0.6780	613	0.0023	0.5923
	DEGseq	9326	0.9297	0.9592	9081	0.9035	0.9494	8812	0.8748	0.9384
	NBPSeq	1860	0.1252	0.7333	1299	0.0707	0.6622	792	0.0277	0.5423
	Bayseq	728	0.0066	0.6686	653	0.0032	0.634	540	0.0008	0.5332
	TSPM	7387	0.3374	0.7515	3005	0.2575	0.6879	1931	0.1524	0.5593
										
V	DESeq	686	0.0084	0.6103	611	0.0050	0.5663	494	0.0018	0.4779
	Edger	747	0.0107	0.6507	667	0.0062	0.6109	549	0.0020	0.5315
	DEGseq	8763	0.8685	0.9454	8609	0.8520	0.9411	8354	0.8247	0.9312
	NBPSeq	1049	0.0431	0.6612	878	0.0293	0.6142	648	0.0139	0.5226
	Bayseq	667	0.0055	0.6186	598	0.0026	0.5746	493	0.0006	0.4886
	TSPM	2072	0.1488	0.7324	1617	0.1047	0.6747	1072	0.0575	0.5544
										
VI	DESeq	789	0.0086	0.6816	679	0.0051	0.6331	547	0.0018	0.5305
	Edger	825	0.0106	0.7301	741	0.0060	0.6869	615	0.002	0.5975
	DEGseq	8811	0.8721	0.9618	8675	0.8576	0.9567	8436	0.8318	0.9493
	NBPSeq	1070	0.0380	0.7283	910	0.0256	0.6790	689	0.022	0.5788
	Bayseq	722	0.0055	0.6727	646	0.0023	0.6254	535	0.0005	0.5310
	TSPM	1920	0.1277	0.7711	1485	0.0086	0.7067	950	0.0418	0.5736
										
VII	DESeq	233	0.0125	0.1211	144	0.0080	0.0725	53	0.0034	0.0220
	Edger	200	0.0121	0.0913	109	0.0070	0.0457	34	0.0026	0.0106
	DEGseq	8429	0.8346	0.9184	8255	0.8163	0.9091	7949	0.7840	0.8929
	NBPSeq	253	0.0170	0.0991	153	0.0108	0.0562	59	0.0047	0.0165
	Bayseq	156	0.0054	0.1076	70	0.0021	0.0508	7	0.0002	0.0047
	TSPM	296	0.0024	0.0571	266	0.0236	0.0537	219	0.0191	0.0476

In Table 3, we summarized the average AUC-ROC for each scenario. For each scenario, DEGseq has less satisfying performance compared to the other methods. Under scenarios II, V, and VII, the average AUC-ROC of baySeq was the largest. Those three scenarios were simulated for the dataset with small sample size or small treatment effects. This indicates that baySeq is the best performing method when the dataset has a small sample size or small treatment effect. Under other scenarios (i.e. I, III, IV, VI), the best performance was generally obtained by edgeR and DESeq, followed by NBPSeq and TSPM.

**Table 3:** Average area under ROC curve (AUC-ROC) under different scenarios

		AUC						
		**I**	**II**	**III**	**IV**	**V**	**VI**	**VII**
		
Methods	DESeq	0.9284	0.7717	0.9097	0.9281	0.8921	0.9326	0.7427
	Edger	0.9322	0.7608	0.9213	0.9323	0.9001	0.9393	0.7336
	DEGseq	0.6081	0.6046	0.6413	0.5722	0.6031	0.6131	0.6000
	NBPSeq	0.9129	0.7559	0.9209	0.8496	0.8828	0.9206	0.7330
	Bayseq	0.9280	0.7843	0.9013	0.9240	0.9013	0.9297	0.7660
	TSPM	0.8744	0.7341	0.9109	0.7688	0.8505	0.8870	0.7176

We also measured the runtime for each method running on the TCGA breast cancer dataset. On a Dell 3500 with a 2.8GHz CPU and 12 GB RAM, DESeq took the most time to finish with over 2 hours followed by NBPSeq (77 minutes), baySeq (47 minutes). TSPM and edgeR took significantly less time in comparison with 6 and 2 minutes, respectively. DEGseq was the speediest of the six, finishing in only 12 seconds. Combining information from both runtime and AUC, edgeR performed best with relatively short runtime and good AUC-ROC.

## Conclusions

In this study, we systematically evaluated six read count-based RNAseq analysis methods using both real and simulated data. BaySeq is the most unique method out of the six, because it is based on an empirical Bayesian approach which produces no fold change or test statistics to infer the direction of the gene expression difference. This inconvenient feature of baySeq forces users to go back to the raw expression value to determine the direction. Another unique characteristic of baySeq is the randomization steps involved in its model. For the same data with the same parameter settings, baySeq produces slightly different results. Granted, genes with the strongest fold change will always be detected by baySeq, but this minor inconsistency between runs of baySeq does produce some inconvenience. From our simulation study, we found that the performances of DESeq, edgeR, and NBPSeq are similar in most cases. This result is expected because all three methods are based on a negative binomial model and differ principally in the way the dispersion parameter is estimated.

One of the issues we observed with these six read count-based RNAseq analysis methods is that they tend to be over-sensitive. For a traditional microarray study, the number of differentially expressed genes identified by a simple method such as a t-test is highly dependent on the sample size. We have tested these six methods using smaller sample sizes such as 2 vs. 2, but the majority of the methods still produced huge amounts of differentially expressed genes (sometimes over 50% of total genes), which is a clear sign of over-sensitivity. Through more thorough analysis, we found that the five methods (excluding baySeq) produced very similar fold changes but less similar ranks of p-value. Given these facts, we would recommend using the combined criteria of fold change and p-value to filter out more false positives. Combined with evaluation of runtime from real data and AUC-ROC from simulated data, edgeR achieved a good balance between speed and accuracy.

We are far from reaching a consensus on the best RNAseq analysis approach. Several unique characteristics of RNAseq data contribute to the difficulty of RNAseq data analysis. The value for non-expression in RNAseq is zero, which means there are no reads aligned to the gene. In microarray, there is always background intensity for non-expressed genes, thus we can always take log transformations of the intensity data. In contrast, due to the large number of zeros in RNAseq data (often more than 50%), we cannot take a log transformation. The range of RNAseq data is between 0 and 50,000, compared to microarray's 2 to 15 after RMA normalization. This huge range of RNAseq data can result in many false high fold changes. Also, there are many sequencing and alignment artifacts that can skew RNAseq data. For example, GC content plays an important factor in the sequenceability of a gene, and the exon's length has a large effect on alignment accuracy. To date, only 1 RNAseq package, DESeq takes the paired data information into consideration. In summary, even though there are a large number of RNAseq analysis tools at our disposal, given the uniqueness of RNAseq data and the number of unsolved problems, there is still much room left to improve RNAseq analysis.

## Competing interests

The authors declare that they have no competing interests.

## Authors' contributions

Yan Guo and Chung-I performed the analysis and wrote the manuscript. Fei Ye and Yu Shyr provided significant scientific input. All authors read and approved the final manuscript.

## Declarations

The publication fee of this manuscript is provided by Harold L. Moses Chair in Cancer Research fund, Vanderbilt University.

This article has been published as part of *BMC Genomics *Volume 14 Supplement 8, 2013: Selected articles from the International Conference on Intelligent Biology and Medicine (ICIBM 2013): Genomics. The full contents of the supplement are available online at http://www.biomedcentral.com/bmcgenomics/supplements/14/S8.
